# Temperature dependent dynamics of DegP-trimer: A molecular dynamics study

**DOI:** 10.1016/j.csbj.2015.04.004

**Published:** 2015-04-28

**Authors:** Nivedita Rai, Amutha Ramaswamy

**Affiliations:** Centre for Bioinformatics, School of Life Sciences, Pondicherry University, Puducherry 605014, India

**Keywords:** DegP-trimer, PDZ1, PDZ2, LA loop, Molecular dynamics simulation, Principal component analysis

## Abstract

DegP is a heat shock protein from high temperature requirement protease A family, which reacts to the environmental stress conditions in an ATP independent way. The objective of the present analysis emerged from the temperature dependent functional diversity of DegP between chaperonic and protease activities at temperatures below and above 28 °C, respectively. DegP is a multimeric protein and the minimal functional unit, DegP-trimer, is of great importance in understanding the DegP pathway. The structural aspects of DegP-trimer with respect to temperature variation have been studied using molecular dynamics simulations (for 100 ns) and principal component analysis to highlight the temperature dependent dynamics facilitating its functional diversity. The DegP-trimer revealed a pronounced dynamics at both 280 and 320 K, when compared to the dynamics observed at 300 K. The LA loop is identified as the highly flexible region during dynamics and at extreme temperatures, the residues 46–80 of LA loop express a flip towards right (at 280) and left ( at 320 K) with respect to the fixed β-sheet connecting the LA loop of protease for which Phe46 acts as one of the key residues. Such dynamics of LA loop facilitates inter-monomeric interaction with the PDZ1 domain of the neighbouring monomer and explains its active participation when DegP exists as trimer. Hence, the LA loop mediated dynamics of DegP-trimer is expected to provide further insight into the temperature dependent dynamics of DegP towards the understanding of its assembly and functional diversity in the presence of substrate.

## 1 Introduction

Every living organism has special mechanisms to respond against a variety of stress conditions such as temperature, pH, and oxidative stress. The heat shock proteins are a group of evolutionarily conserved proteins which respond to stress conditions by protein folding/degradation in the presence of ATP [1,2]. HtrA (high temperature requirement protease A) is one such heat shock protein, which fulfils these roles without consuming ATP. It is a widely conserved protein lying on the extra-cytosolic compartment of prokaryotes as well as eukaryotes. There are various homologues of HtrA found in *Escherichia coli* such as DegP, DegQ and DegS [3,4]. DegP is an essential protein of *E. coli* which behaves as molecular chaperone and protease under stress condition in a temperature dependent manner for cell survival. It is experimentally reported that below 28 °C, DegP behaves as chaperone, but efficiently degrades unfolded protein above 28 °C [5,6].

DegP shares a common structural architecture with all the HtrA proteins which comprises serine protease and PDZ (*P*ostsynaptic density protein/*D*lg1/*Z*o1) domains. A mature DegP monomer has 448 amino acid residues in which residues 1–259 form N-terminal serine protease domain and two PDZ domains at the C-terminal (namely PDZ1 and PDZ2) are formed by residues 260–358 and 359–448, respectively [7]. The protease domain also contains (i) catalytic triad formed by Ser210, His105 and Asp135 residues and (ii) activation loops: LD (168–175), L1 (205–209), L2 (227–238), L3 (185–198) and LA (36–81) [8,9]. The LA loop of DegP contains a disulphide bridge between residues Cys57 and Cys69, which makes the structure comparatively compact and reduction of this S–S bond leads to autocatalysis of DegP [10,11]. The N-terminal protease domain is not only required for the protease activity but also plays a vital role in chaperonic activity, whereas the C-terminal domain involves in substrate interaction and DegP oligomerization [12]. The protease domain is connected with PDZ1 domain by a flexible loop region where Arg262 and Gly263 act as a hinge and play a key role in changing the orientation of PDZ1 domain. The PDZ2 domain is connected to PDZ1 via flexible linker residues Ser357–Ser364. Both these linkers are essential for protease activity of hexameric DegP [13].

DegP is a multimeric protein that exists in active and inactive forms [14]. The minimal functional unit of DegP is a trimer which is capable of performing both protease and chaperonic activities [15]. This DegP trimer (hereafter referred as DegP-trimer) is stabilized by the interaction between the protease domains of adjacent monomers. DegP exists in an inactive state when two trimeric units form a hexamer. In this hexameric form, the LA loop of each monomers in one trimeric subunit interacts with L1, L2 and LD loops of other trimer and blocks the catalytic site due to which, the hexameric unit adopts resting/inactive state. In this resting/inactive state, DegP exists either in open or closed form [16]. In the open form, the two trimeric units are connected only via the LA loops and provide a wide lateral passage for the substrate binding. Whereas, in the closed form, in addition to the LA loop interaction (as observed in open form), there is cross-talk between the PDZ domains of the two trimeric units, i.e., the PDZ1 of one trimeric unit interacts with the PDZ2 of other trimeric subunit [17–19]. As a consequence of these domain rearrangements, the proteolytic site is completely shielded from the solvent [13]. The protease and PDZ1 domains govern the protease activity while the protease domain alone is sufficient to express chaperonic activity [15]. The PDZ1 domain of DegP contains a deep substrate binding hydrophobic cleft referred as E–L–G–I pocket (similar to G–L–G–F motif of serine protease family) formed by the carboxylate binding loop residues from 264 to 267 [20]. When substrate binds to the inactive hexamer, the hydrophobic cleft of PDZ1 domain recognizes the C-terminal residues of the substrate and places it into the inner cavity formed by the protease domain [21–23]. This binding signal is then transmitted to the activation loops (L1, L2 and LA) via the sensory L3 loop and mediates the activation of hexamer by dissociating into transient trimeric units. Subsequently, the trimers assemble into catalytically active higher order oligomers such as 12-mer and 24-mer. These oligomers are reverted to the inactive hexameric form after the completion of its function [24].

Molecular dynamics simulation methods have been successfully applied in exploring the structural and functional aspects of various bio-molecules including conformational transition/diversity in nanosecond timescale [25–28]. Various studies on temperature dependent simulations have demonstrated its ability to explore the functional diversity of biomolecules with respect to temperature [29–31]. Principal component analysis (PCA) is a useful technique in analysing the trajectories from molecular dynamics simulations. PCA transforms a number of possibly correlated variables into a smaller set of linear variables and this reduction in dimension gives low frequency independent subspace, called as essential subspace, in which the functionally relevant motions occur [32,33]. The total mobility of a system is described by the sum of eigenvalues, in which the first few eigenvectors with higher percentage of variance describe the collective dynamics of the molecule. Hence, the PCA of molecular dynamics trajectory effectively differentiates the low frequency collective motions and high frequency localized motions [34,35]. Recently, the allosteric regulation of HtrA2 by PDZ domain is well documented using molecular dynamics simulations [36]. In this present work, the structural dynamics of DegP-trimer is studied at various temperatures like 280, 300 and 320 K using molecular dynamics simulations. The present study is a first report explaining the structural dynamics of DegP-trimer with special emphasis on the effect of temperature using computational methods.

## 2 Materials and methods

### 2.1 Modelling of DegP-trimer

The crystallographic structure of DegP deposited in the Protein Data Bank (PDB ID: 1KY9) was used for the present study. DegP contains 448 amino acid residues, out of which, the structure of residues forming regulatory loop regions (Asp52–Gly78 and Ser188–Tyr195) and other residues like Gly370–Ala374 and Met447–Gln448 are not reported. Structure of the LA loop region (Asp52–Gly78) was predicted using ab-initio structure prediction tool Quark [37] and the other missing residues were modelled using SPDBV v4.0.1 software [38]. The monomeric coordinates were replicated using VMD software according to the symmetry reported in crystallization studies to model the structure of DegP-trimer [39]. This modelled and validated DegP-trimer was used for further molecular dynamics simulation studies.

### 2.2 Molecular dynamics simulations

MD simulations of DegP-trimer at various temperatures (280, 300 and 320 K) were performed in GROMACS 4.5.5 for a period of 100 ns using GROMOS43a1 force field [40]. The structure of DegP-trimer was solvated using SPC/E water model extending 12 Å from the extents of DegP and was neutralized by replacing six water molecules with Cl^−^ ions. The system was initially relaxed using the steepest descent algorithm followed by conjugate gradient algorithm and the minimization was automatically truncated when the force is lesser than 1000.0 kJ/mol/nm. The long range electrostatic interactions were evaluated using the Particle Mesh Ewald method [41] with a cut-off of 10 Å. All non-bonded interactions were treated by the Lennard–Jones interaction with a cut-off of 10 Å. The bonds involving hydrogen atoms were constrained using LINCS algorithm [42]. Constant temperature was maintained using V-rescale thermostat for NVT ensemble with a coupling constant of 0.1 ps [43]. The Parrinello–Rahman Barostat for NPT ensemble was used at a constant pressure of 1 bar, coupling constant of 2.0 ps and compressibility of 4.5e − 5/bar [44]. The entire system was equilibrated using NVT and NPT ensembles for 500 and 100 ps, respectively. Finally, the system was simulated without any constraints using NPT ensemble for a period of 100 ns to understand the temperature dependent structural dynamics of DegP-trimer.

#### 2.2.1 Cross correlation analysis

The cooperative domain motions of DegP-trimer were analysed using the correlation matrix *R_ij_* generated for the Cα-atoms (N = 1314) ranging between − 1 and 1 [45,46]. The extent of displacement between the residues is calculated by correlation coefficient of each pairs of Cα atoms *i* and *j* as given below.

$$R_{ij}=\;\frac{\langle \Delta r_{i}\;\cdot \Delta r_{j}\rangle }{\sqrt{\langle \Delta r_{i}^{2}\rangle \langle \Delta r_{j}^{2}\rangle }}$$

where, Δ*r_i_* and Δ*r_j_* are the displacements from the mean position of *i*th and *j*th of Cα atoms and are averaged over the entire trajectory. Cross-correlation matrix is generated by using cpptraj module of Amber Tools 13 [47].

#### 2.2.2 Principal component analysis

PCA is one of the convenient methods to examine the structural evolutions explored by a MD trajectory. PCA is based on the covariance matrix, which captures the degree of co-linearity of atomic motions describing the internal dynamics. The principal components (PCs) are obtained by the orthogonal transformation of protein Cartesian coordinates [48]. The covariance matrix (*C_ij_*) is calculated from the mass-weighted Cartesian coordinates (*i* and *j*) of the N-particle system sampled over the simulated trajectory and is given by

$$C_{ij}=\langle \left(x_{i}-\langle x_{i}\rangle \right)\left(x_{j}-\langle x_{j}\rangle \right)\rangle \text{.}$$

The covariance matrix *C_ij_* is diagonalized by an orthogonal coordinate transformation matrix *R* to get the diagonal matrix with eigenvalues *λ*

$$R^{T}C_{ij}R=I\lambda \text{.}$$

Here, *R* represents the eigenvectors or principal modes and *I* is an identity matrix of dimension 3N and *λ* represents eigenvalues. Each eigenvalue is associated to an eigenvector, which gives the direction of the new coordinate. The projection of eigenvectors with respect to these eigenvalues gives the PCs (*p*_*i*,_ where *i* = 1, …, 3N).

$$p=R^{T}\left(x-\langle x\rangle \right)$$

Here, the eigenvalue *λ* denotes the mean-square fluctuation in the direction of respective principal mode for the DegP-trimer having 1314 Cα-atoms with 3942 eigenvectors.

## 3 Results and discussion

**Fig. 1:**
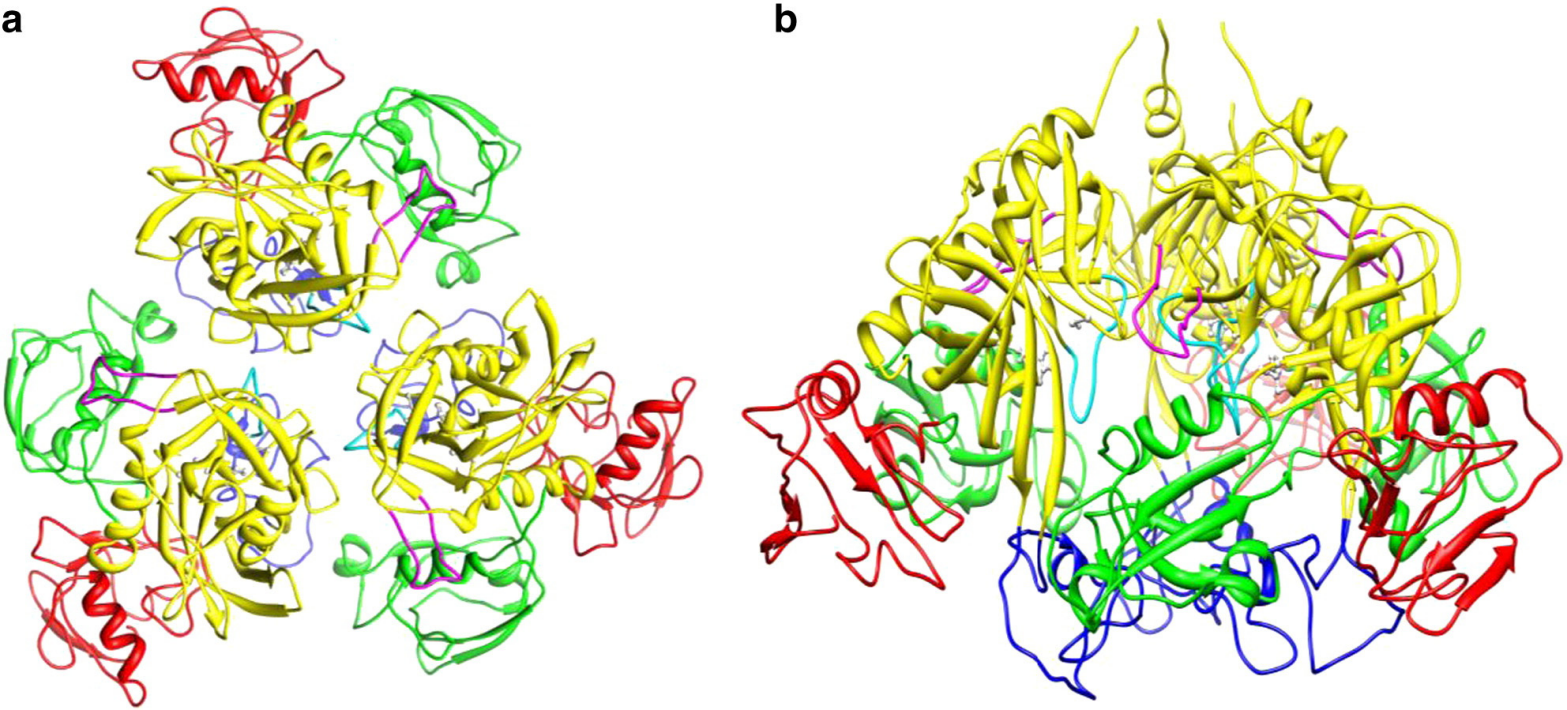
The structure of modelled DegP-trimer in top (a) and side (b) views. The LA, L2 and L3 loops are coloured as blue, cyan and magenta, respectively and the rest of protease domain is coloured as yellow. The domain PDZ1 is in green colour and the PDZ2 is coloured red.

The structure of DegP-trimer was modelled and validated using the Ramachandran plot in which 84.2% residues come under the most favoured regions and 13.8% residues are present in additionally favoured regions. The modelled structure of DegP-trimer is shown in Fig. 1. All atom simulations performed on the modelled DegP-trimer for a period of 100 ns were analysed systematically. It is reported that DegP expresses chaperonic activity at temperature below 28 °C and involves in protease activity above 28 °C [49]. In line with this report, the structural dynamics of DegP-trimer was studied at various temperatures (280, 300 and 320 K) to elucidate the temperature dependent structural aspects behind the functional diversity of DegP.

**Fig. 2:**
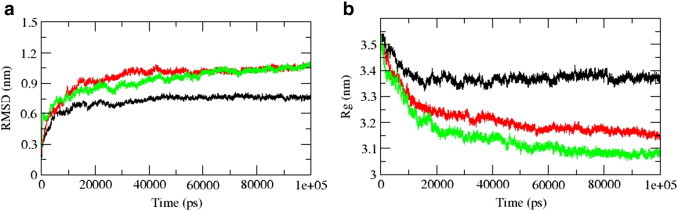
The variation in backbone RMSD (a) and radius of gyration of Cα-atoms (b) of DegP-trimer observed during dynamics at 280 (black lines), 300 (red lines) and 320 K (green lines), respectively.

The root mean square deviation (RMSD) calculated for the backbone of DegP-trimer during the simulation of 100 ns is shown in Fig. 2(a). In the simulations performed at 280, 300 and 320 K, DegP-trimer expressed stable dynamics after 20 ns and hence, the analyses of DegP-trimer have been performed on the trajectories observed between 20 and 100 ns. DegP-trimer at 280 K expresses stable dynamics with RMSD about 0.75 nm, whereas, at both 300 and 320 K, it expressed comparatively increased dynamics with RMSD closer to 1.15 nm. The RMSD of individual monomers of DegP was examined further to analyse such higher RMSD. Supplementary Fig. 1 clearly explains the RMSD of protease domain (with and without LA loop region), PDZ1 and PDZ2 domains as well. The analysis revealed that the domains of DegP monomers are highly stable during dynamics and the observed higher RMSD is a consequence of LA loop dynamics as well as inter-monomeric interactions.

The root mean square deviation (RMSD) calculated for the backbone of DegP-trimer during the simulation of 100 ns is shown in Fig. 2(a). In the simulations performed at 280, 300 and 320 K, DegP-trimer expressed stable dynamics after 20 ns and hence, the analyses of DegP-trimer have been performed on the trajectories observed between 20 and 100 ns. DegP-trimer at 280 K expresses stable dynamics with RMSD about 0.75 nm, whereas, at both 300 and 320 K, it expressed comparatively increased dynamics with RMSD closer to 1.15 nm. The RMSD of individual monomers of DegP was examined further to analyse such higher RMSD. Supplementary Fig. 1 clearly explains the RMSD of protease domain (with and without LA loop region), PDZ1 and PDZ2 domains as well. The analysis revealed that the domains of DegP monomers are highly stable during dynamics and the observed higher RMSD is a consequence of LA loop dynamics as well as inter-monomeric interactions.

The compactness of DegP-trimeric assembly is monitored (Fig. 2(b)) by calculating the radius of gyration (Rg). The DegP-trimer at 280 K is highly stable with a Rg value of 3.38 nm and is assembled more closely at both 300 and 320 K due to which a decrease in Rg value (3.17 and 3.07 nm, respectively) was observed. All these observations ensure a temperature dependent change in the configuration of DegP-trimer.

### 3.1 Intra- and inter-monomeric interactions in DegP-trimer

The stability of DegP-trimer, governed by the cooperative and anti-cooperative intra- as well as inter-monomeric motions, is studied by plotting the cross-correlation matrices. Fig. 4 shows the correlated motions observed in DegP-trimer at various temperatures such as 280 (a), 300 (b) and 320 K (c), in which each monomer is highlighted by a box. The positive regions indicate cooperative motions between residues, where the strength of correlation increases from yellow to red (scaled between 0.1 and 1.0). The negative regions coloured in blue (scaled between − 0.6 and − 0.1) are defined by the anti-cooperative domain motions. The uncorrelated regions separating both correlated and anti-correlated are coloured in cyan (from − 0.1 to 0.1).

DegP-trimer expresses strong intra- as well as inter-monomeric motions. The strong cooperative motions observed in DegP-trimer at 280 K are between: (i) LA loop of monomer 1 and PDZ1 (from Arg262 to Ala322 except Gln292 to Ala302) of monomer 2, and (ii) LA loop of monomer 2 with both LA loop and PDZ1 of monomer 3. In DegP trimer, the protease domain in all three monomers shares a strong cooperative motion among themselves. The LA loop of monomer 1 is anti-cooperative with the PDZ2 of monomer 2. The PDZ2 of monomer 2 shares a strong anti-cooperative motion with the PDZ1 of monomer 3. In general, it is also observed that all three monomers express a similar pattern of correlated and anti-correlated inter- as well as intra-domain motions at 280 K.

At 300 K, the prominent anti- cooperative motion of LA loop with respect to its protease domains is not expressive as observed at 280 K. Similarly, the anti-cooperative motion of PDZ2 of monomer 1 against the protease and PDZ1 of monomer 2 is also not expressed at 300 K. DegP-trimer ensures an inter-monomeric cooperative interaction of the LA loop of monomer 1 with the PDZ1 of monomer 2. In addition, the protease and PDZ2 (which moves cooperatively) of monomer 2 share a cooperative motion with the protease domain of monomer 1. The PDZ2 of monomer 2 moves together with both PDZ1 and PDZ2 domains of monomer 3. The amplitude of correlation is significantly diminished at 300 K when compared to the observed correlation at 280 K.

The anti-cooperative motions are marginally more pronounced when DegP-trimer is heated to 320 K. The monomer 1 involves in a strong intra-domain interactions, except the LA loop, which significantly expresses an anti-correlated motion with the rest of its domains. The protease (except LA loop) and PDZ1 of monomer 1 express a significant anti-cooperative motion with both PDZ1 and PDZ2 domains of monomers 2 and 3. These strong anti-cooperative motions promote the LA loop to adopt a new conformation during dynamics. The cooperative motion of both protease and PDZ1 domains at 320 K shows their role in protease activity, which is not observed with the simulation at 280 K.

The observed coordinated motions of LA loop with various parts of DegP and its monomeric units emphasize its active participation for the functional dynamics of DegP-trimer. The coordinated motions observed between protease domains also ensure their role in maintaining the assembly of DegP-trimer. All these observations evidence temperature dependent domain motions between the monomeric units of DegP-trimer.

### 3.2 Dynamics of LA loop

The LA loop, which is an important regulatory element of DegP-trimer, expresses dynamic behaviour and effectively participates in both intra- and inter-monomeric domain motions. The LA loop is highly networked by ~ 22 intra-LA H-bonds and is consistent irrespective of the temperature during simulation. The H-bonds between LA loop and other interacting domains of the monomeric units were also examined. At room temperature, the LA loop is networked with more number of inter-H-bonds (i.e., 8 ± 2) and is comparatively free at 280 and 320 K with lesser inter-H-bonds (3 ± 1 and 4 ± 1, respectively) due to its flipping motion from the protease core.

**Fig. 3:**
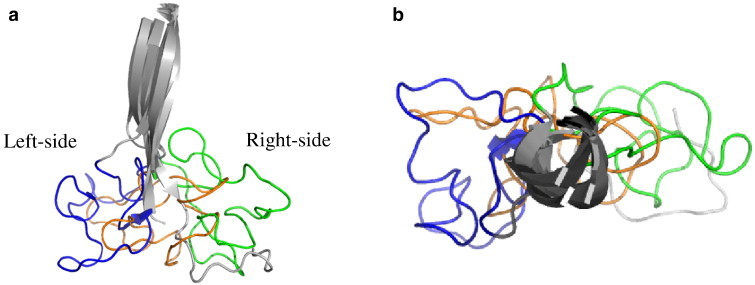
Temperature dependent dynamics of LA loop displayed in side view (a) and top view (b) of monomer 1 simulated at 280, 300 and 320 K along with the cryo-EM (PDB ID: 2ZLE) structure and are coloured as green, orange, blue and grey, respectively.

**Fig. 4:**
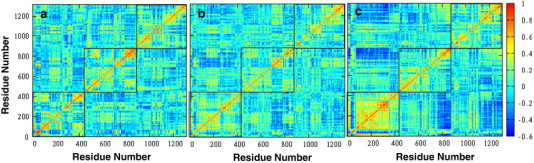
The correlative motions between the Cα-atom of residues of DegP-trimer observed during the last 80 ns of simulation performed at 280 (a), 300 (b) and 320 K (c), respectively. Each monomeric unit is outlined by a box. The amplitude of correlation varies from blue (anti-cooperative region) to red (highly cooperative region) via the uncorrelated region coloured as cyan.

Analysis on the temperature dependent dynamics of LA loop revealed variable conformations with respect to temperature and is shown in Fig. 3. During simulations, the LA loop residues Thr36–Thr39, Arg41–Arg44, Gln48, and Gly72–Gln81 form instantaneous H-bond with the neighbouring domains of the same monomer. Simulations at 320 K revealed a transformation of this highly flexible LA loop from the initial conformation to a new conformation, i.e., at 320 K, the region of LA loop encompassed by residues 46–80 is completely flipped off from its initial position and reoriented towards its PDZ2 domain and the PDZ1 domain of the neighbouring monomeric unit. Precisely, this flip is towards the left side (indicated in Fig. 3) with respect to the fixed β-sheets that connect the LA loop of protease domain. To identify the residues responsible for this transformation at 320 K, the dihedral angles (φ and ψ) of residues Phe46–Phe50 and Gly78–Gln82 (those forming the neck of the flipped region) were monitored. It is clear that the dihedral angle of these residues shows significant variation at 320 K, i.e., the φ (ψ) angle of residues Phe46, Gln80 and Gln81 varies in the range − 129 to − 179° (− 139 to − 104), − 176 to − 159 (40 to 176) and − 177 to − 166 (− 179 to − 180), respectively. The residues 47–49 modulate their dihedral angles according to the variation in the dihedrals observed for Phe46 to facilitate the transition.

At 280 K, there is no such flipping of LA loop as observed at 320 K. The LA loop expresses stable dynamics around the initial resting position and communicates interactions with its PDZ1 domain (green ribbon in Fig. 3). This conformation of LA loop is closer to the conformations observed from the cryo-EM method (PDB ID: 2ZLE), when the DegP exists in chaperonic conformation (grey ribbon in Fig. 3). This observation evidences its ability in adopting similar conformation (favouring chaperonic activity) even when DegP exists as trimer.

Similar to the moderate interactions revealed by the correlation map (at 300 K), the LA loop is not involved in any conformational flip but is stabilized in between the two conformations observed at 280 and 320 K. Overall, the present analysis discloses a temperature sensitive dynamics of LA loop and promotes further insight into the dynamics of DegP-trimer.

### 3.3 Principal component analysis

**Fig. 5:**
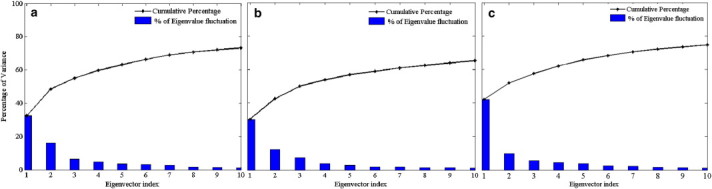
Percentage (blue bars) and cumulative percentage (black line) of variance for the first 10 eigenvalues obtained from the covariance matrix of Cα atoms observed during the last 80 ns simulations performed at 280 (a), 300 (b) and 320 K (c), respectively.

The last 80 ns of the simulated trajectories of DegP-trimers was used to perform PCA. Fig. 5 shows the plot of the first ten eigenvalues resolved from the covariance matrix of fluctuations in decreasing order against the eigen-indices for DegP-trimer. Comparison of eigenvalues indicates that the first ten principal components account more than 65% of the global motion (73.27, 65.22 and 74.81% at 280, 300 and 320 K, respectively). It is observed that PC1 contributes 32.46, 30.36 and 42.26% for the motion, whereas the PC2 accounts 16.1, 12.24 and 9.74% for the motion at 280, 300 and 320 K, respectively. It is also visible that both magnitude and percentage of contribution of eigenvalues at 280 and 320 K for PC1 are higher than that of simulation at 300 K and evidences the presence of a single major essential subspace for the structural dynamics of DegP-trimer at lower and higher temperatures. In addition, it is clear that the first eigenvalue contribute significantly to the global motions when compared to the rest of eigenvalues that diminishes quickly as they correspond to the localized fluctuations.

**Fig. 6:**
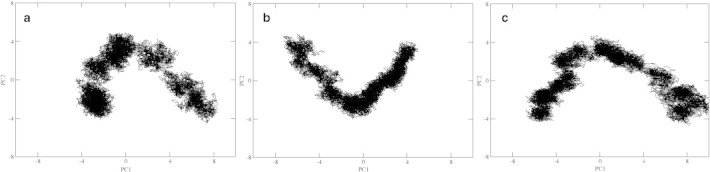
Projection of Cα-atom trajectories on the first two eigenvectors (in nm) of DegP-trimer simulated at 280 (a), 300 (b) and 320 K (c), respectively.

In PCA, the direction of motion is extracted by projecting the trajectory observed between 20 and 100 ns over the desired eigenvector to understand the dynamics of protein in the direction of respective eigenvector. Fig. 6 depicts the projection of first two PCs to display the motions of DegP-trimer in a 2-dimensional subspace. It provides a measure of mobility where each cluster represents the tertiary conformational changes observed along the trajectory. At 280 K, the DegP-trimer spans in a larger conformational subspace, while at 300 K, it spans in a comparatively smaller with lesser amplitude and reveals a comparatively confined motion of DegP-trimer. At 300 K, the direction of motion is exactly reverse when compared to the direction of motion observed at 280 and 320 K. When temperature increases to 320 K, the DegP-trimer adopts similar pattern of motion as observed at 280 K, but with increased amplitude in the direction of PC1. These plots ensure that DegP-trimer exists in a structurally active state at higher and lower temperatures than the room temperature.

### 3.4 Residue fluctuations in PC1

**Fig. 7:**
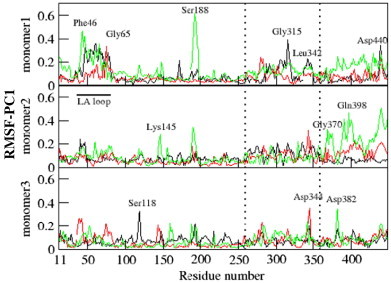
Residue displacements (in nm) in the subspace spanned by the first eigenvector of DegP-trimer at 280 K (black), 300 K (red) and 320 K (green).

Fig. 7 shows the displacement of residues corresponding to the motion described by first PC at 280, 300 and 320 K, respectively. It is observed that all the monomers show variation in their amplitude of motion. DegP possesses many dynamic regions such as LA, L2 and L3 loops, and PDZ1 and PDZ2 domains. Among these regions, the LA loop (Phe46 to Gly65) of DegP expresses higher fluctuation. Such higher fluctuation of LA loop explains its temperature dependent mobility. At higher temperature (i.e., 320 K), the L3 loop, which acts as a signalling region, expresses higher fluctuation in all the monomers and indicates its participation during dynamics. The PDZ1 domain motion is comparatively moderate at 320 K and few residues like Gly370, Asp382, Gln398, and Asp440 of PDZ2 domain show higher fluctuations. At 280 K, apart from LA loops, few more residues like Ser118, Gly315, Leu342 and Asp440 express fluctuation. The residues of sensory loop L3 (Ser188, Gly189 and Glu193) that transmits the substrate binding signal to activation domain are highly triggered by high temperature [16,50,51].

In DegP-trimer, the secondary structures like β-barrel and β-sheets are involved in stable dynamics throughout the simulation. In general, the residue fluctuations at 280 and 320 K are highly pronounced when compared to that observed at 300 K and explain the dual nature of DegP-trimer at extreme (higher and lower) temperatures and the passive role of DegP-trimer at room temperature.

### 3.5 Domain motions in DegP-trimer

**Fig. 8:**
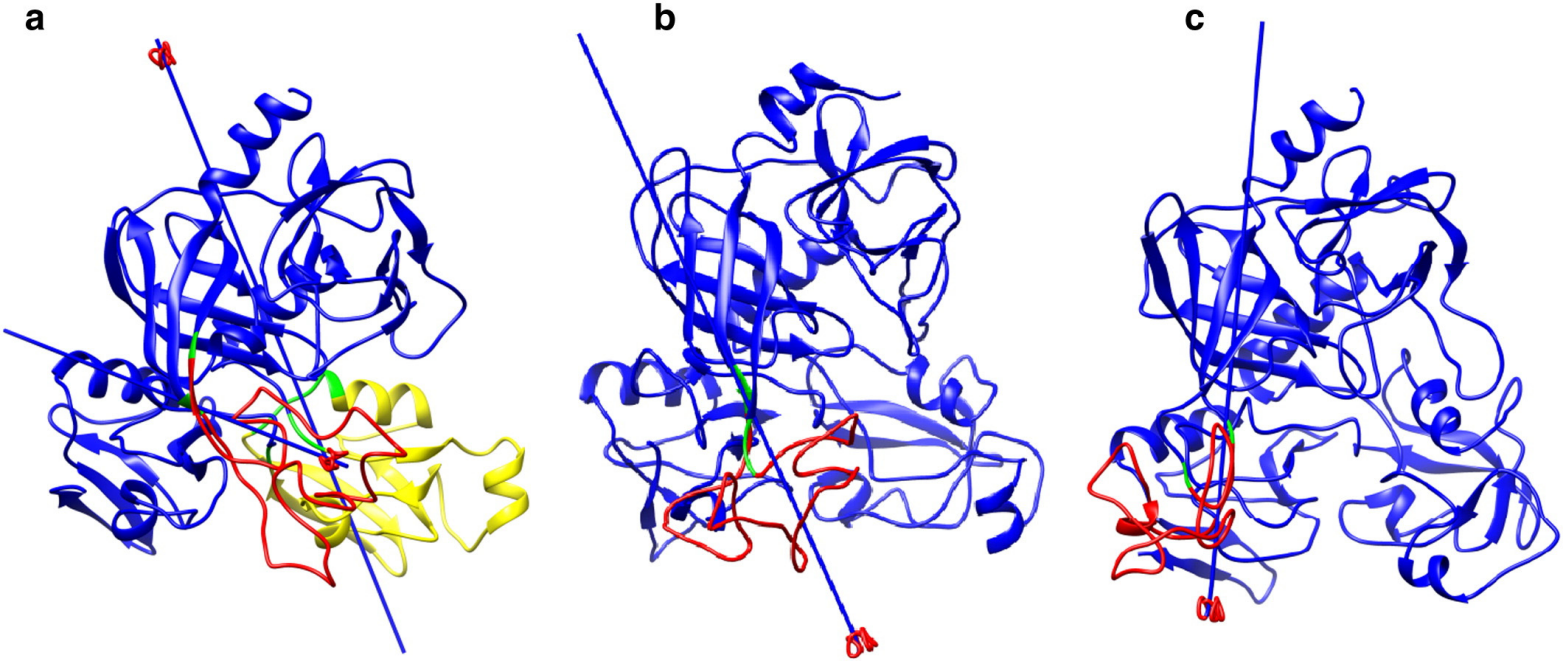
Domain motions described by the monomer from DegP-trimer at 280 (a), 300 (b) and 320 K (c) temperatures respectively. The fixed and moving domains are coloured in blue, red, and yellow respectively. The axis of rotation of moving domain with respect to the fixed domain is also depicted.

The domain motions have also been analysed using DynDom programme [52] to quantify the translational cum rotational dynamics of individual domains (Fig. 8 and Table 1). At 280 K, the residues Ala16–Ser34 and Met85–Gln259 of protease, Val260–Gly263, Thr330–Val333, Leu354–Ser358 of PDZ1 and Gln359–Leu446 of PDZ2 domains are defined as the fixed domain with respect to which the translational cum rotational motions of the moving domains are explained. DegP possesses two moving domains defined by (i) Thr36–Phe81 of LA loop including Thr35, Gln82–Phe84 of protease domain (referred as moving domain 1) and (ii) Glu264–Gly329 and Val333–Glu353 of PDZ1 domain (referred as moving domain 2) for which the identified hinge residues are (i) Ser34–Thr35, and Phe84–Met85 and (ii) Gly263–Glu264, Gly329–Val333, and Glu353–Leu354, respectively. Both of the moving domains 1 and 2 express rotational (of about ~ 57°) cum translational motion (of 0.3 and 2.1 Å, respectively) with respect to the fixed domain. It is also observed that, the substrate binding hydrophobic loop (E–L–G–I motif) is identified as the hinge region for these motions. Both LA loop and PDZ1 moving domains express a closure of 41.3 and 24.9%, respectively with respect to the fixed domain. As the degree of closure measures the closeness of respective moving domains towards the fixed domain, the observed 41.3% closure of LA loop towards the PDZ1 domain indicates that at lower temperature (i.e. at 280 K), the LA loop moves closure to the chaperonic conformation.

At 300 K, the residues Thr36–Gln80 are observed as moving domain with respect to the fixed domain formed by Pro17–Gln259 of protease, Val260–Ser358 of PDZ1 and Gln359–Thr442 of PDZ2 domains, for which the residues of LA loop (Ser34–Thr35 and Gln80–Gln81) act as hinge. The moving LA loop expresses a translational (− 4.6 Å) cum rotational (58.8°) motion with 66.9% of closure property.

At 320 K, the only identified moving domain (Thr36–Gly78 of LA loop) expressed rotational (82.4°) cum translational (− 3.7 Å) motion with 61.3% of closure with respect to the fixed domain. The residues Pro13–Thr35 and Gly79–Gln259 of protease along with both PDZ domains form the fixed domain and the residues Thr36–Gly78 serve as the hinge residues. From the DynDom analysis of DegP-trimer at 280, 300 and 320 K, it is clear that DegP at 300 K exists in a comparatively intermediate state, while at lower and higher temperatures, DegP adopts two different conformations, which discriminate the dual function of DegP. The axis of rotation also supports the flip of LA loop to both the right and left sides of the anti-parallel beta sheet for the functional diversity of DegP-trimer.

It is also hypothesized that the signals are transmitted consecutively rather than simultaneously; an asymmetry is induced in the structure [51]. Our present analysis also reveals a structural asymmetry during dynamics, i.e. high amplitude motion is expressed by one LA loop and the interacting L3 loop, when compared to the dynamics of LA and L3 loops in other monomeric units. In general, all these observations reveal a dynamics structural state of DegP-trimer like other oligomer and also a temperature dependent dynamics of LA loop, which plays a key role in determining the function of DegP.

## 4 Conclusion

In this present analysis, the structure of DegP-trimer was built based on the reported crystal symmetry. Molecular dynamics simulations at various temperatures 280, 300 and 320 K were performed on DegP-trimer for 100 ns, to understand the temperature induced structural dynamics. Under the influence of temperature, both molecular dynamics as well as PCA analyses revealed a stable dynamics of DegP-trimer by relaxing the tertiary structures while maintaining the secondary structures. The stability of DegP-trimer is highly mediated by both intra- as well as inter-monomeric motions. Specifically, the LA and L3 loop regions and PDZ1 domain contribute significantly to the dynamics of DegP. At room temperature, DegP-trimer exists in a passive state with less dynamic property. Simulations at 280 and 320 K expressed pronounced dynamics of DegP-trimer. At 280 K, the LA adopts a conformation closer to the conformation observed when DegP functions as chaperone, whereas the LA loop at 320 K flips with respect to Phe46 in a direction opposite to the flip at 280 K and such dynamics might coordinate the signalling between the neighbouring monomers. It is reported that the phenylalanine residues present at the core region of LA loop play a key role in promoting the flexible dynamics of LA loop according to the temperature shift [53]. Our observation of Phe46 as one of the key residues in promoting the conformational changes of LA loop also reinforces its role in dynamics. Even though, all the three monomers are involved in the functional dynamics of DegP-trimer, only one monomer takes the lead in expressing high amplitude motions of LA loop in association with temperature shift.

In general, it is interesting to observe the temperature induced high amplitude dynamics of DegP-trimer (via loop regions and PDZ1 domain) at both lower and higher temperatures. The overall observations signify the role of flexible LA loop over the structural dynamics of DegP-trimer which might promote further understanding on the temperature dependent functions of DegP. Various intriguing aspects on DegP mechanism like fate of substrate and transition between different oligomeric states are yet to be explored to further understand the function of DegP. In line with these observations, the structural dynamics of DegP-hexamer in the presence of substrate is in progress to understand the allosteric activation mechanism of DegP from the structural point of view.

The following are the supplementary data related to this article.

Supplementary Fig. 1 RMSD of protease with LA (black), without LA (red), PDZ1 (green) and PDZ2 (blue) extracted from simulation of DegP-trimer at 280 K (a), 300 K (b) and 320 K (c).

Supplementary data to this article can be found online at http://dx.doi.org/10.1016/j.csbj.2015.04.004.

## Figures and Tables

**Table 1:** Relative motions of the moving domains with respect to the fixed domain.

Relative domain motions	280 K		300 K	320 K
Fixed domain	Protease: 16–34, 85–259 PDZ1: 260–263, 330–333, 354–358 PDZ2: 359–446		Protease: 17–34, 81–259 PDZ1: 260–358 PDZ2: 359–442	Protease: 13–35, 79–259 PDZ1: 260–358 PDZ2: 359–446
Moving domain 1	Protease: 35, 82–84 LA loop: 36–81		LA loop: 36–80	LA loop: 36–78
Moving domain 2	PDZ1: 264–329, 333–353		Nil	Nil
Angle of rotation (°)	LA loop terminal	PDZ1	58.8	82.4
	57.7	56.0		
Translation along axis (Å)	0.3	2.1	− 4.6	− 3.7
Closure (%)	41.3	24.9	66.9	61.3
Bending residue	34–35, 84–85	263–264, 329–333, 353–354	34–35, 80–81	35–36, 78–79
