# Supplementary material for: Temperature dependent dynamics of DegP-trimer: A molecular dynamics study
Source: Comput Struct Biotechnol J. 2015 Apr 28;13:329–38. doi: 10.1016/j.csbj.2015.04.004 (PMC4434178; doi:10.1016/j.csbj.2015.04.004)
Supplement: Supplementary Fig. 1 — RMSD of protease with LA (black), without LA (red), PDZ1 (green) and PDZ2 (blue) extracted from simulation of DegP-trimer at 280 K (a), 300 K (b) and 320 K (c). [file mmc1.docx]

**Supplementary Figure 1**: RMSD of protease with LA (black), without LA (red), PDZ1 (green) and PDZ2 (blue) extracted from simulation of DegP-trimer at 280 K (a), 300 K (b) and 320 K (c).


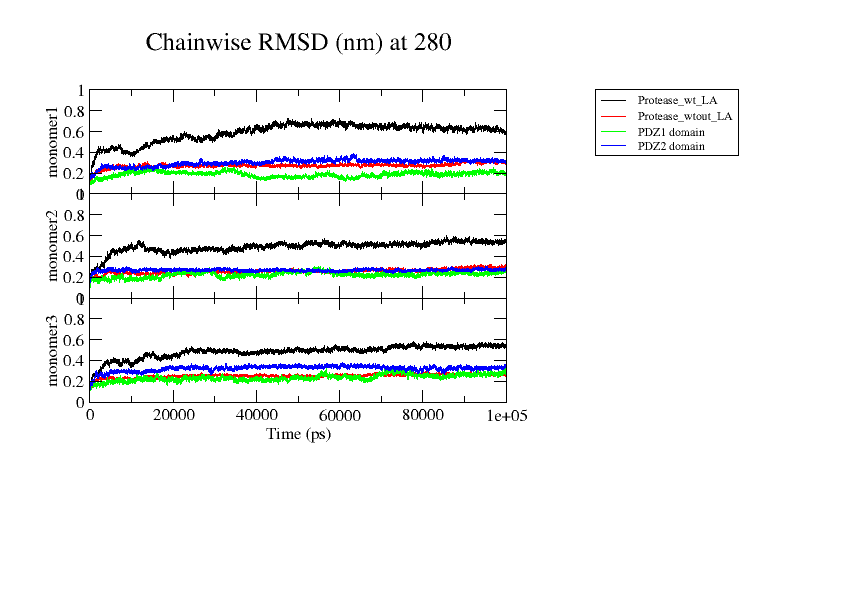


**ab**


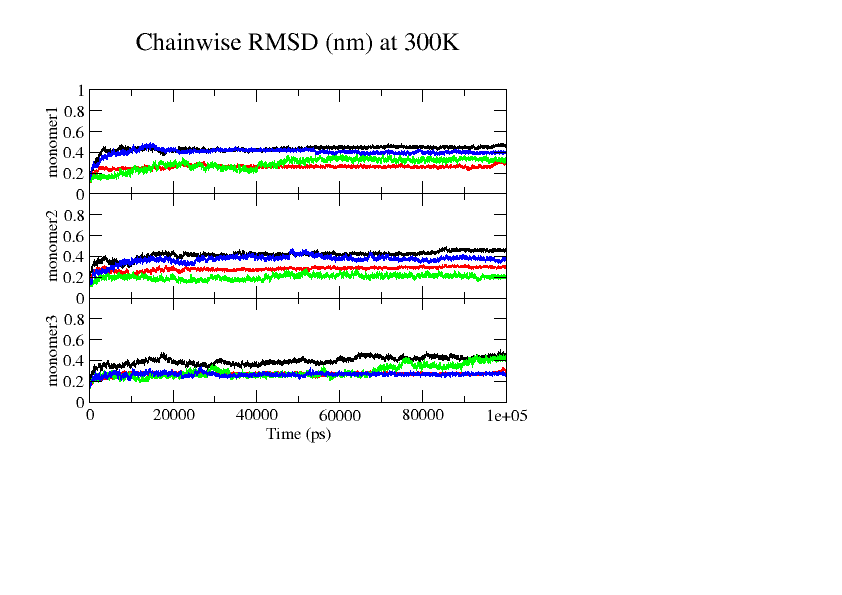


**b**


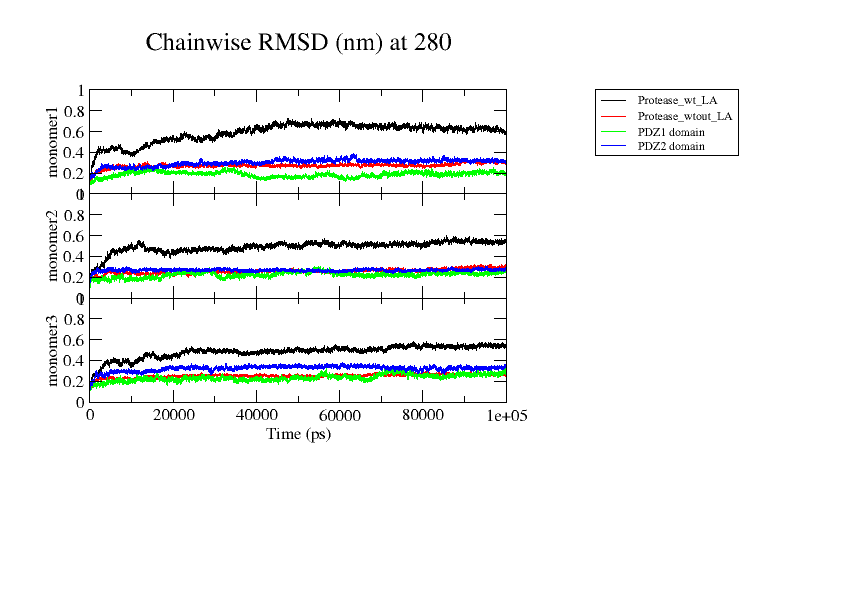

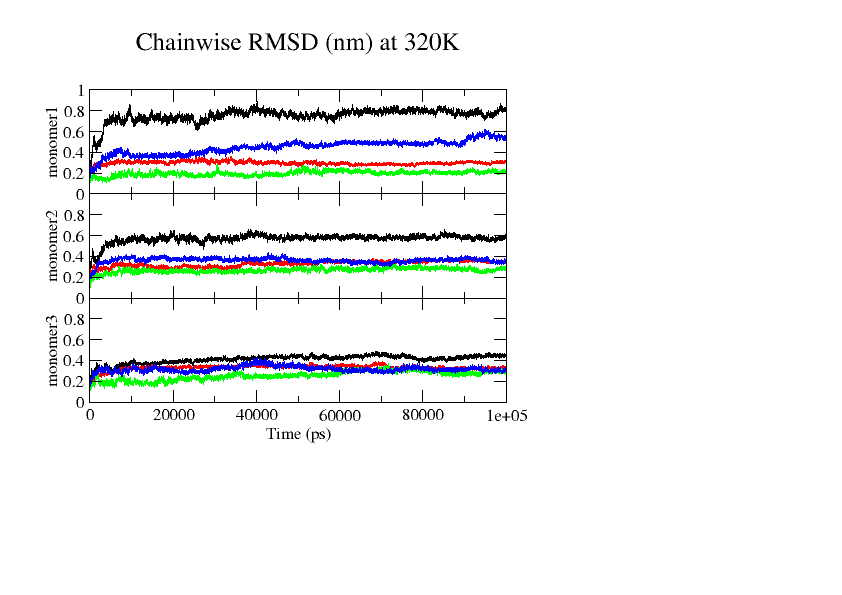


**c**


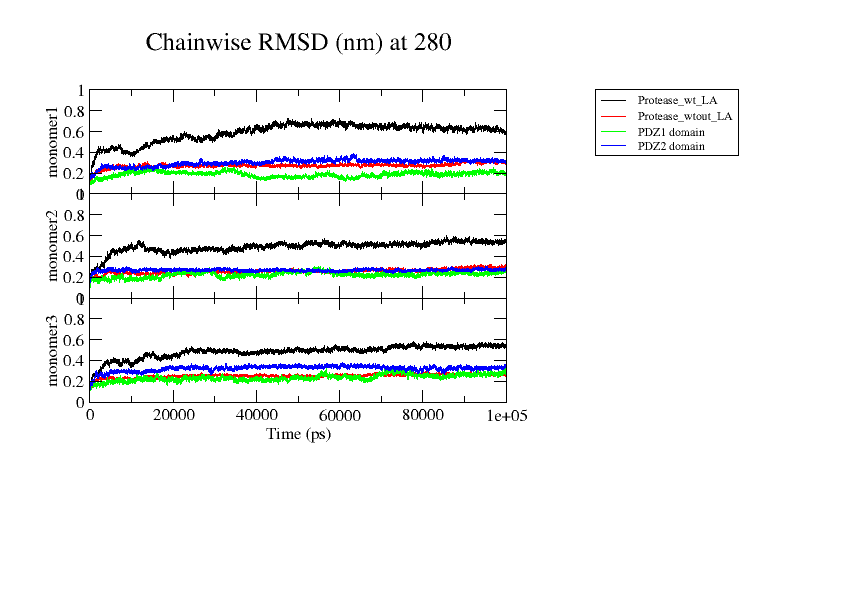


**RMSD (nm)**

**RMSD (nm)**

**RMSD (nm)**
